# Influence of the Polymer Glass Transition Temperature and Molecular Weight on Drug Amorphization Kinetics Using Ball Milling

**DOI:** 10.3390/pharmaceutics12060483

**Published:** 2020-05-27

**Authors:** Camilla Asgreen, Matthias Manne Knopp, Jeppe Skytte, Korbinian Löbmann

**Affiliations:** 1 Department of Pharmacy, University of Copenhagen, DK-2100 Copenhagen, Denmark; camilla.asgreen@sund.ku.dk; 2 Pharmacosmos A/S, DK-4300 Holbaek, Denmark; jeppe.skytte@gmail.com; 3 Bioneer:FARMA, Department of Pharmacy, University of Copenhagen, DK-2100 Copenhagen, Denmark; matthias.manne.knopp@sund.ku.dk

**Keywords:** amorphous, solid dispersion, ball-milling, milling time, amorphization kinetics

## Abstract

In this study, the putative correlation between the molecular mobility of a polymer and the ball milling drug amorphization kinetics (i.e., time to reach full drug amorphization, *t*_a_) was studied using different grades of dextran (Dex) and polyvinylpyrrolidone (PVP) and the two model drugs indomethacin (IND) and chloramphenicol (CAP). In general, IND had lower *t*_a_ values than CAP, indicating that IND amorphized faster than CAP in the presence of the polymers. In addition, an increase in polymer molecular weight (M_w_) also led to an increase in *t*_a_ for all systems investigated up to a critical M_w_ for each polymer, which was in line with an increase of the glass transition temperature (T_g_) up to the critical M_w_ of each polymer. Hence, the increase in *t*_a_ seemed to correlate well with the T_g_/M_w_ of the polymers, which indicates that the polymers’ molecular mobility had an influence on the drug amorphization kinetics during ball milling.

## 1. Introduction

The use of the amorphous form is one of the most promising approaches to overcoming the solubility challenge presented by the increasing amount of poorly soluble drugs [1,2,3]. Since amorphous solids are disordered and thus in a higher energy state compared to their crystalline counterparts [1], they are thermodynamically unstable and tend to crystallize during storage. Hence, stabilization of the amorphous form, especially through incorporation in a polymeric carrier to create a so-called amorphous solid dispersion (ASD), have been widely investigated [3]. Since the drug itself may be miscible or even soluble in the polymer, such an ASD can in theory be thermodynamically stable if the drug loading is below its equilibrium solubility in the polymer at the storage conditions [4]. In contrast, drug loadings above the equilibrium solubility will eventually lead to the crystallization of the drug [5]. For these thermodynamically unstable systems, other factors that contribute to the stabilization of the amorphous drug in an ASD become more relevant, such as potential intermolecular interactions between the drug and polymer [6], or a decrease in molecular mobility [7].

In this regard, it has been shown that increasing the polymer chain length/molecular weight (M_w_) increases the physical stability of an ASD of indomethacin (IND) in polyvinylpyrrolidone (PVP) as a result of overall reduced molecular mobility [8]. Furthermore, the M_w_ of the polymer has also been shown to affect the dissolution rate and performance of the drug from an ASD. Here, increasing the M_w_ of PVP decreased the dissolution rate of celecoxib compared to PVP with lower M_w_ [9]. Nevertheless, the ASDs with the lower M_w_ PVP did not display the best overall dissolution performance (area under the dissolution-time curve) due to an inferior precipitation-inhibiting effect compared to the ASDs containing higher M_w_ PVP.

In order to incorporate the drug into the polymer network, several methods are described in the literature, including ball milling, quench cooling and spray drying. Ball milling is often used in early-stage research as it can be performed at a small scale and does not require any heat or solvents [10]. On the other hand, the method requires a milling time long enough for the incorporation of the drug into the polymer network to achieve full amorphization, which may be as long as 2 h when using an oscillatory ball mill or even 8 h when using a planetary mill [10,11]. However, little is known about the influence of polymer M_w_ on the ball-milling time required to reach full drug amorphization (*t*_a_). In this study, it is hypothesized that the incorporation of a drug into the polymer is dependent on the molecular mobility of the polymer. Hence, polymers with higher M_w_ may require longer milling times to obtain an ASD during milling. In this regard, it has been shown that the relationship between the increasing glass-transition temperature (T_g_) and the increasing M_w_ of a polymer only applies up to a critical M_w_, above which the T_g_ does not increase further [12,13]. Since the T_g_/M_w_ of a polymer is also indirectly a measure of the molecular mobility of the polymer, this study aims to investigate the putative correlation between the molecular mobility (T_g_/M_w_) of a polymer and the ball-milling amorphization kinetics (*t*_a_) of different grades of dextran and PVP, and the two model drugs IND and chloramphenicol (CAP).

## 2. Materials and Methods

### 2.1. Materials

Dextrans of different M_w_ (kDa) (Dex 1, Dex 1.5, Dex 3.5, Dex 5, Dex 10, Dex 12, Dex 40, Dex 70 and Dex 500) were kindly supplied by Pharmacosmos A/S (Holbaek, Denmark), and PVPs of different M_w_ (K12, K17, K30 and K90) were kindly supplied by BASF (Ludwigshafen, Germany). PVP K60 and chloramphenicol (CAP) were sourced from Sigma Aldrich (St. Louis, MO, USA) and indomethacin (IND) was sourced from Fagron (Copenhagen, Denmark).

### 2.2. Differential Scanning Calorimetry

To determine the T_g_ of the different grades of PVP, a Discovery differential scanning calorimeter (DSC) from TA Instruments Inc. (New Castle, DE, USA) was used. Samples (2–4 mg) were analysed in TA Instruments Tzero aluminium hermetic pans with a perforated lid under 50 mL/min nitrogen gas flow using modulated temperature DSC (mDSC). The samples were heated from 5–200 °C using a heating rate of 2 °C/min with a modulation amplitude of 0.212 °C over a period of 40 s. The T_g_ (midpoint) was determined using the TA Instruments TRIOS software (version 4.1.1).

### 2.3. Ball Milling

The time to reach full drug amorphization (*t*_a_) was studied using an oscillatory ball mill (Mixer mill MM400, Retsch GmbH & Co., Haan, Germany), which was placed in a cold room (5 °C). Physical mixtures of drug and polymer (1000 mg) were placed in 25 mL jars containing two 12 mm stainless steel ball bearings (Retsch GmbH & Co., Haan, Germany) and milled at a frequency of 30 Hz. The investigated drug-polymer ratios were 10:90 *w*/*w* and 50:50 *w*/*w* for mixtures containing dextrans and PVP, respectively. In order to obtain homogeneous mixtures, the drug-polymer powder mixtures were mixed without the ball bearings for 5 min (0 min sample). Subsequently, the ball bearings were added to the jars and samples of approximately 10 mg were collected at predetermined time points, from 0–60 min. Furthermore, both IND and CAP were milled without polymer to evaluate their amorphization kinetics against the drug-polymer mixtures.

### 2.4. X-Ray Powder Diffraction

The samples collected from ball milling were analysed using X-ray powder diffractometry (XRPD) with an X’Pert Pro diffractometer from PANalytical (Almelo, the Netherlands) in order to evaluate whether they were X-ray amorphous. The diffractometer operated in reflection mode with the use of CuKα radiation (1.54187 Å), with the acceleration voltage and current set to 45 kV and 40 mA, respectively. Samples were scanned from 5 to 30° 2θ on aluminum sample holders with a scan speed of 0.067° 2θ/s and a step size of 0.026°. Results were analyzed using the X’Pert Data Viewer software (version 1.2, PANalytical, Almelo, the Netherlands). The amorphousness of the samples in this study was investigated solely using XRPD, which does not provide information on the homogeneity of the sample, i.e., whether a homogeneous single-phase amorphous solid dispersion has been created after the applied *t*_a_. Hence, the time *t*_a_ refers to the samples being X-ray amorphous.

## 3. Results

**Figure 1:**
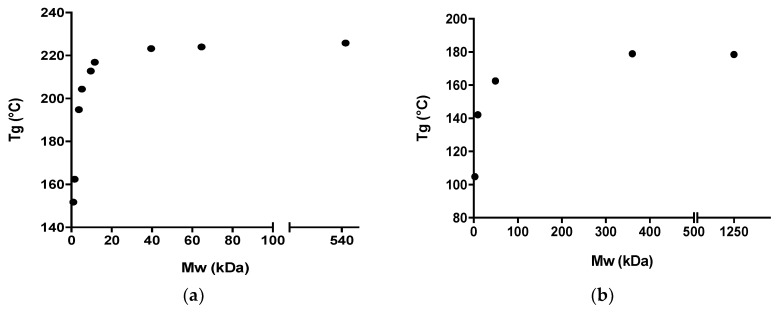
Correlation between the T_g_ (°C) and the M_w_ (kDa) for (**a**) different dextran grades and (**b**) different PVP grades. The T_g_ values for the different dextran grades were obtained from [13].

It has previously been shown that the T_g_ of dextrans increases with increasing M_w_ up to around 40 kDa, after which a further increase in M_w_ did not result in a significant further increase in the T_g_ [13]. Similarly, the T_g_ of PVP only increased with increasing M_w_ up to around 360 kDa (see Table 1 and Figure 1).

The pure drugs were milled in order to investigate their behaviour upon milling for 60 min. It was found that pure IND fully amorphized after 60 min of milling (*t*_a_ = 60 min). Hence, the drug can be prepared in its amorphous form without a carrier. On the other hand, pure CAP does not fully amorphize even after 60 min of milling, which was evident in the remaining crystalline diffractions in the XRPD diffractograms (data not shown). CAP is therefore not able to amorphize on its own under these conditions.

**Figure 2:**
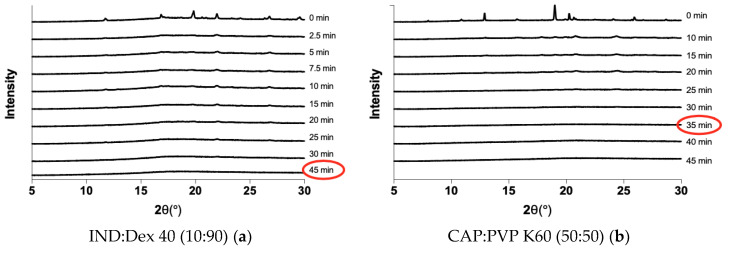
X-ray powder diffractometry (XRPD) diffractograms showing the decrease in crystallinity over time for a mixture of IND and Dex 40 (10:90 *w*/*w*) (**a**) and a mixture of CAP and PVP K60 (50:50 *w*/*w*) (**b**). The *t*_a_ is marked with a red circle.

The amorphization kinetics upon milling were investigated for the different drug-polymer systems. The *t*_a_ was characterized as the first time point where no Bragg peaks were visible in the XRPD diffractograms. Table 1 summarizes the *t*_a_ values for the different systems, and two representative examples for the amorphization kinetics are shown in Figure 2. Figure 2a illustrates that IND fully amorphized in the presence of Dex 40 after 45 min of milling, as indicated by the lack of Bragg peaks in the diffractogram. Figure 2b illustrates that it was also possible to obtain an ASD containing CAP and PVP K60 with a *t*_a_ of 35 min.

**Figure 3:**
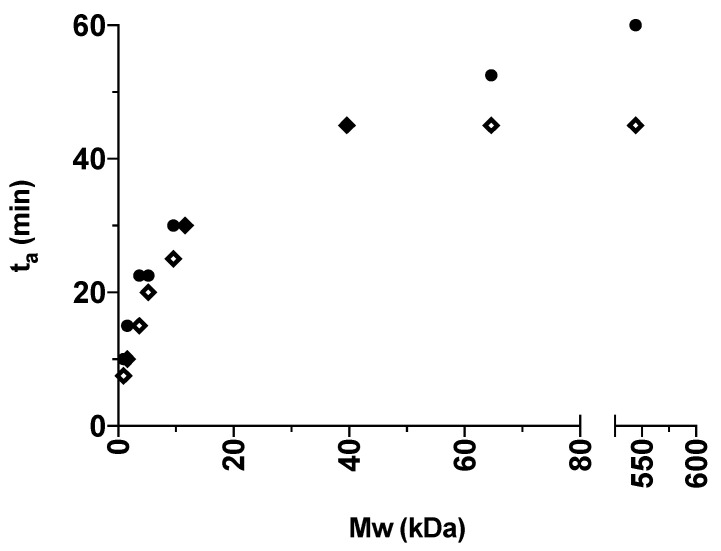
The *t*_a_ (min) of the different IND:Dex 10:90 *w*/*w* (diamond) and CAP:Dex 10:90 *w*/*w* (circle) mixtures plotted against the average M_w_ (kDa) of the polymer grade in the certain mixture.

**Figure 4:**
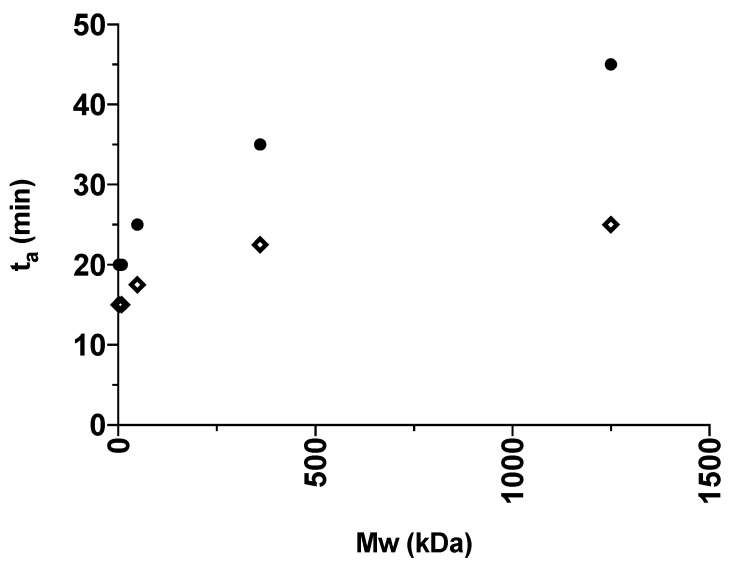
The *t*_a_ (min) of the different IND:PVP 50:50 *w*/*w* (diamond) and CAP:PVP 50:50 *w*/*w* (circle) mixtures plotted against the average M_w_ (kDa) of the polymer grade in the certain mixture.

For a better visualization, the obtained *t*_a_ values presented in Table 1 are illustrated in Figure 3 and Figure 4 for the various grades of dextrans and PVP, respectively. In general, IND had lower *t*_a_ values than CAP, indicating that IND amorphized faster than CAP in the presence of the polymers. This is in line with the findings from the milling of the pure drugs, where IND could be amorphized on its own. In addition, an increase in polymer M_w_ also led to an increase in *t*_a_. The fastest amorphization kinetics were obtained for Dex 1 and PVP K12 (i.e., the polymers with the lowest M_w_). With the increasing M_w_ of the polymers, the subsequent increase in *t*_a_ is most pronounced for dextrans grades below 40 kDa (Dex 40) and PVP grades below 360 kDa (PVP K60). Furthermore, the presence of the polymers allowed CAP to become amorphous and reduced the *t*_a_ of IND compared to milling the drugs on their own.

When comparing Figure 1 with Figure 3 and Figure 4, it can be seen that an increase in M_w_ affects both the T_g_ of the polymer and the *t*_a_ in a similar fashion. Furthermore, the increase of both *t*_a_ and T_g_ is most pronounced for lower polymer M_w_, and less pronounced after the critical polymer M_w_ is reached. Hence, this finding indicates that the polymer M_w_ (i.e., molecular mobility) appears to influence the amorphization kinetics, and hence the *t*_a_. In this regard, it is important to emphasize that it has been shown that the polymer M_w_ does not influence the equilibrium solubility of the drug in the polymer at ambient temperature [14]. On the other hand, it has been shown that an ASD comprising the drug IND together with PVP of a higher M_w_ (K30) showed increased physical stability as a result of overall reduced molecular mobility compared to an ASD with PVP of lower M_w_ (K12) [8]. In other words, whilst a higher M_w_ is more efficient at preventing crystallization, it also requires longer milling times to achieve a fully amorphous solid dispersion.

Finally, it should be noted that, apart from the T_g_/M_w_ of the polymer, there are potentially other factors contributing to the overall amorphization kinetics. This study showed for example that the good glass former IND showed faster amorphization kinetics than the poor glass former CAP when being milled together with a given polymer. It is also known that the polymer M_w_ does not influence the equilibrium solubility of the drug in the polymer [14]. Nevertheless, in light of the equilibrium solubility of the drug in the polymer, it would be interesting to investigate how different drug loadings would impact the amorphization kinetics. In this context, molecular interactions between the drug and the polymer, the strength of these interactions and the final T_g_ of the formed ASD all potentially contribute to the amorphization kinetics during milling. Lastly, the presence of water in a polymer significantly impacts its properties, as water acts as a plasticizer [15], lowering the polymer’s T_g_ and increasing its mobility, but also reducing the equilibrium solubility of the drug in the polymeric carrier [16]. Hence, given that most polymers are hygroscopic, the impact of water represents another critical parameter to be investigated in future studies.

## 4. Conclusions

In summary, this study found a correlation between the polymer T_g_/M_w_ and ball-milling amorphization kinetics. The findings support our original hypothesis that increasing polymer M_w_ will increase the *t*_a_, probably as a consequence of decreased molecular mobility.

## Figures and Tables

**Table 1:** The polymer molecular weight (M_w_) and corresponding glass-transition temperature (T_g_) for dextrans (Dex) and polyvinylpyrrolidone (PVP) and their influence on the ball milling time to reach full drug amorphization (*t_a_*) for both chloramphenicol (CAP) and indomethacin (IND).

Compound	M_w_ (kDa)	T_g_ (°C)	*t*_a_ (min)	
			CAP	IND
Dex 1	1.0 ^a^	151.8 ± 0.2 ^c^	10.0	7.5
Dex 1.5	1.6 ^a^	162.4 ± 1.1 ^c^	15.0	10.0
Dex 3.5	3.7 ^a^	194.8 ± 0.4 ^c^	22.5	15.0
Dex 5	5.2 ^a^	204.4 ± 0.8 ^c^	22.5	20.0
Dex 10	9.6 ^a^	212.8 ± 0.9 ^c^	30.0	25.0
Dex 12	11.6 ^a^	216.9 ± 0.3 ^c^	30.0	30.0
Dex 40	39.6 ^a^	223.2 ± 0.0 ^c^	45.0	45.0
Dex 70	64.6 ^a^	224.0 ± 0.6 ^c^	52.5	45.0
Dex 500	544.3 ^a^	225.8 ± 0.8 ^c^	60.0	45.0
PVP K12	2.5 ^b^	104.8 ± 0.9	20.0	15.0
PVP K17	9.0 ^b^	142.1 ± 0.7	20.0	15.0
PVP K30	49 ^b^	162.5 ± 0.2	25.0	17.5
PVP K60	360 ^b^	178.9 ± 0.6	35.0	22.5
PVP K90	1250 ^b^	178.5 ± 0.2	45.0	25.0

^a^ Average Mw (Mn) provided from Pharmacosmos, ^b^ Average Mw (Mn) provided from BASF, ^c^ Data from [13].
